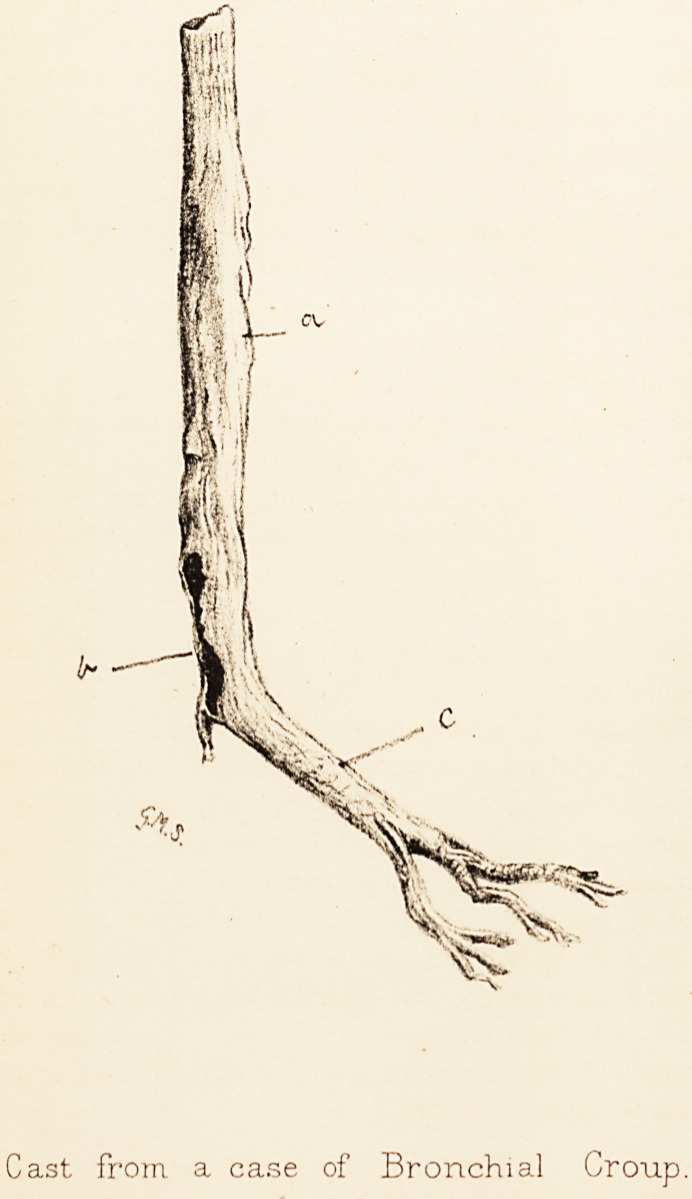# Notes on a Case of Bronchial Croup

**Published:** 1886-06

**Authors:** G. Arthur Brown

**Affiliations:** Tredegar


Cast from a case of Bronchial Croup.

### NOTES ON A CASE OF BRONCHIAL CROUP.

By G. Arthur Brown, Tredegar.

The specimen, of which a drawing is annexed, is a fibrinous cast of the trachea, and its continuation down the left bronchus. The tracheal portion (*a*) of the cast measures three and a quarter inches in length, and seven-sixteenths of an inch in diameter. It forms a hollow tube, the wall of which is thin and fragile, but which after hardening in spirit is fairly firm and resistent. At its lower end is a long ragged opening (*b*) about one inch in vertical length, from which the continuation down the right bronchus appears to have been torn. The lining of the left bronchus (*c*) is complete, measuring two inches, and of smaller diameter than the tracheal portion. At its lower extremity it divides dichotomously, the first divisions having a length of three-quarters of an inch, and then dividing again; the second divisions of the bronchial lining again being bisected after another three-quarters of an inch in length. There are, accordingly, indications of divisions to the fourth degree from the trachea down the bronchial tubes.

The following is an outline of the case:

A. L., aged 17, a dressmaker, attended chapel on the evening of Sunday, the 25th of April. She returned home complaining of feeling cold and ill. She passed a restless night, and on the following morning, the 26th, I was called to see her. The patient had been known to me for the last twelve or thirteen years as a delicate girl. I had frequently during that period attended her for bronchial attacks, and once for pneumonia. In January last she suffered from a mild attack of scarlatina, from which she made a good recovery.

Her father—who was an asthmatic subject, and long suffered from emphysema of the lungs—died of pneumonia two years ago. Three years ago, her eldest sister died of phthisis. Her brother and two surviving sisters are distinctly strumous subjects. Her mother is a stout but unhealthy woman.

On visiting the patient I found her dressed, and lying on a sofa. She complained of a feeling of tightness about the chest, and of an irritable cough with no expectoration. Her respiration was quiet (19) but slightly croupy; temperature 99.5°; skin hot and dry; tongue slightly coated and moist. There was no exudation visible about the fauces or glottis then or at any period of the case. There was no dulness on percussion over the lungs. On auscultating the chest I found the respiratory sounds roughened, but there were no moist sounds to be heard over any part. I prescribed an expectorant mixture containing nitrate of potass, acetate of ammonia, antimonial wine (10 minims) and ipecacuanha, to be taken every four hours. I also ordered her an aperient, and gave the general directions applicable to the management of what appeared to be a case of incipient bronchitis.

On the following day, the 27th, the patient expressed herself as feeling better. The skin was moist, but there was still no expectoration; the cough was less troublesome, but the husky inspiration, and the sounds heard on auscultation, were but little altered.

On the 28th the temperature had risen to 101°, and the feeling of constriction was more marked. There was still no expectoration. The pulse was 100, and feeble. Air was freely admitted into all parts of the lungs, but the respiratory sounds in front were distinctly and loudly sibilant. I prescribed a mixture containing 15 grains of chloride of ammonium, 4 grains of iodide of potassium, with 20 drops of ipecacuanha wine and a drachm of spirit of nitrous ether, to be taken every four hours, and directed the chest to be enveloped in linseed poultices, and that the patient should inhale the steam of hot water constantly.

Early in the morning of the 29th she was seized with a violent paroxysm of coughing, and succeeded in expectorating a perfect cast of the trachea and its bifurcation. She was very greatly relieved and passed a good day, the mixture and the inhalations being continued. Towards night the feeling of constriction about the chest returned with increased intensity, the cough was tiresome, and she got but little sleep. There was some slight expectoration during the night, which, however, was not kept for me to see.

Early in the morning of the 30th I was hastily summoned to the patient, and I then found her struggling for breath, her face pinched, her lips blue, and her extremities cold; the pulse was scarcely to be felt. Little or no air was entering the chest, and she said she was dying. The croupy inspiration was very marked, and she implored me to do something to relieve her agony. With little expectation of rendering her much help, but at the earnest solicitation of the patient and her friends, and hoping that possibly I might, by means of a free opening into the trachea, allow of the expulsion of the membrane, I performed tracheotomy. Little or no relief was afforded by the operation, which I fear I was scarcely justified in performing, and the patient shortly sank asphyxiated. No post-mortem examination of the body was permitted.

*Remarks.*—This case appears to me to be one of croupous bronchitis,—more correctly, probably, bronchial croup,—and is unique in my own experience. The fullest account of croupous bronchitis I have met with is contained in the article by Riegel in the fourth volume of Ziemssen's *Cyclopædia,* and the symptoms and course of A. L.'s case accord closely with that writer's description of the complaint in its acute form. He speaks of the disease as being exceedingly rare. "Croupous bronchitis," he says, "as a genuine primary form of disease, is a very rare affection, and one which occurs more frequently in the chronic than in the acute form. Its occurrence is so infrequent, that even in large hospitals years and decades may pass before a single case of the kind comes under observation. Of acute fibrinous bronchitis, with fibrinous expectoration, Lebert could find but seventeen observations after a careful analysis of all the cases known at the time of writing."

Whether the exudation in A. L.'s case commenced in the trachea, and thence extended to the smaller divisions of the bronchi, or *vice versâ,* I am unable to satisfy myself; for until the cast had been expelled, I confess to not having recognised the exact nature of the attack. The absence of the characteristic fibrinous expectoration, which is described as usually occurring in this complaint, and which would have probably cleared up the diagnosis in the earlier period of the case, renders it probable that the deposit may have commenced in the trachea, and thence extended to the finer divisions of the bronchi.

## Figures and Tables

**Figure f1:**